# Characterization and adsorption performance of nano-hydroxyapatite synthesized from *Conus litteratus* waste seashells for Congo red dye removal

**DOI:** 10.1039/d4ra07733d

**Published:** 2024-12-06

**Authors:** Md Sohag Hossain, Md Sahadat Hossain, Samina Ahmed, Mashrafi Bin Mobarak

**Affiliations:** a Institute of Glass and Ceramic Research and Testing (IGCRT), Bangladesh Council of Scientific and Industrial Research (BCSIR) Dhaka-1205 Bangladesh shanta_samina@yahoo.com mashrafibinmobarak@gmail.com

## Abstract

In this research, nano-hydroxyapatite synthesized from *Conus litteratus* seashells (Ss/nHAp) and the potential of Ss/nHAp as an adsorbent for eliminating Congo Red (CR) dye from aqueous solutions were explored. The synthesized Ss/nHAp was subjected to characterization using various techniques, including XRD, XPS, FTIR, Raman, BET, FESEM *etc.* in order to understand the material thoroughly. Batch adsorption experiments were conducted to establish the optimal conditions for removing the dye, considering variables such as adsorbent dosage, contact time, pH and initial dye concentration. 85% of the CR dye was eliminated within a short span of 10 minutes using a minimal adsorbent dose of 0.1 g, under neutral pH and room temperature, showcasing its efficiency. The adsorption process adhered to pseudo second order kinetics and was best fitted by the Langmuir isotherm model, with a maximum adsorption capacity (*q*_max_) of 24 mg g^−1^. The material's reusability was demonstrated through regeneration studies, with efficiency slightly decreasing to 74% by the fifth cycle due to adsorbent loss. This study establishes Ss/nHAp as a low-cost, eco-friendly, and effective adsorbent for dye-contaminated water treatment, paving the way for future applications in industrial wastewater remediation.

## Abstract 2

A schematic representation of the synthesis of nHAp from *Conus litteratus* waste seashells and its application in CR dye removal.

## Graphical abstract

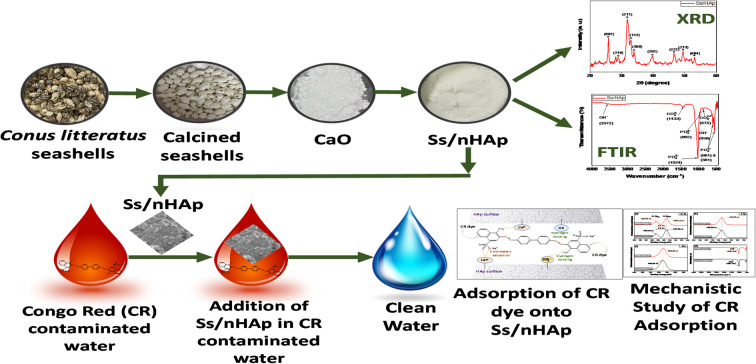

## 1. Introduction

Hydroxyapatite (HAp) is a bioactive material known for its remarkable compatibility with biological tissues, making it an essential component. HAp is widely utilized in various biomedical applications, such as bone grafts, drug delivery, dental implants and tissue engineering scaffolds.^1–3^ The chemical structure of HAp closely resembles the mineral composition found in human bone, which enhances its ability to promote cell attachment and growth.^4^ Beyond its biological significance, HAp is extensively used in the coating of implants to enhance their integration with surrounding tissues and improve their overall functionality.^5^ HAp's versatility lies in its adaptable physical and chemical properties, making it a valuable material in industries beyond healthcare, particularly in environmental remediation.^6,7^

HAp can be synthesized through various methods, broadly classified into dry and wet techniques.^8^ Dry methods, such as solid-state reactions and thermal decomposition, typically involve the mixing of precursor powders, followed by high-temperature treatment to induce chemical reactions.^9^ However, these dry methods come with significant drawbacks, including the need for high temperatures (often exceeding 900 °C), long reaction times, and the risk of particle agglomeration.^10^ These aspects restrict the ability to precisely control the particle size, morphology and crystallinity of the synthesized HAp, which are critical properties for specific applications.^11^

In contrast, wet methods offer several advantages, particularly in controlling the purity, crystallinity, and morphology of the synthesized HAp. Among the wet techniques, chemical precipitation stands out as one of the most efficient and widely used methods for HAp synthesis.^12^ This method typically involves the reaction of calcium and phosphate precursors in an aqueous medium under controlled conditions.^12^ A key benefit of the chemical precipitation method is its ability to be conducted at room temperature and atmospheric pressure, which minimizes the need for expensive equipment and reduces energy consumption. Additionally, this method does not require prolonged reaction times, making it more time-efficient and economically feasible.^4,13,14^ The low energy requirements and ease of controlling the reaction parameters make chemical precipitation an ideal choice for large-scale production of HAp with consistent quality.^15^

Another significant development in HAp synthesis is the use of biogenic materials as raw sources of calcium.^16^ This approach has garnered attention not only due to its economic benefits but also for its positive environmental impact.^17^ Traditionally, calcium sources such as calcium nitrate or calcium hydroxide are used in HAp synthesis.^18^ However, these chemicals can be expensive and environmentally taxing. As a result, researchers have turned to naturally occurring, calcium-rich materials like eggshells, corals, and seashells.^19–22^ These biogenic materials offer an eco-friendly and cost-effective alternative for HAp production, aligning with the principles of green chemistry.^23^

Among biogenic materials, seashells are particularly attractive for HAp synthesis. Composed primarily of calcium carbonate (95–99%), along with minor quantities of oxides and organic materials, seashells are a rich and renewable source of calcium.^24^ The use of waste seashells not only provides an abundant and inexpensive raw material for HAp production but also addresses environmental concerns related to the disposal of seashell waste.^25^ In many coastal areas, including Bangladesh, seashells accumulate as a by-product of the seafood industry, often contributing to environmental pollution. Cox's Bazar, the world's longest sea beach, is an abundant source of seashells, offering a sustainable and readily available resource for industrial applications. Using these discarded seashells for HAp production could significantly reduce the environmental burden while providing an economical source of calcium.

The elimination of hazardous pollutants such as synthetic dyes from industrial wastewater is a major environmental challenge faced by many industries, including textile,^26^ paper,^27^ and leather production.^28^ Congo Red (CR), a commonly used azo dye, is one of the most significant pollutants, especially in the textile sector. CR is known for its high solubility in water, making it challenging to remove utilizing conventional methods of wastewater treatment. Having carcinogenic properties and being harmful to aquatic organisms,^29^ it presents a significant danger to both the environment and human health.^30,31^ Therefore, it is crucial to develop efficient and sustainable methods to remove CR from wastewater prior to releasing it into water sources.

Adsorption is widely recognized as a highly efficient technique for removing dyes from water-based solution.^32,33^ Materials like activated carbon,^34,35^ zeolites,^36^ and biochars^37^ have been extensively studied for this purpose. However, HAp has become as a promising adsorbent in recent years because of its porous structure,^38^ high surface area, and functional groups that can interact with dye molecules.^6^ Additionally, HAp's ability to undergo ion exchange with contaminants makes it particularly suitable for the adsorption of anionic dyes like CR.^39^

In this study, HAp derived from *Conus litteratus* seashells (Ss/nHAp) was synthesized *via* a chemical precipitation method. This method was chosen for its ability to produce high-purity HAp at room temperature without the need for high-pressure or high-temperature conditions. Seashells were selected as the calcium source due to their high calcium carbonate content, availability in Cox's Bazar, and minimal cost, making them an attractive biogenic material for sustainable HAp production. The synthesized Ss/nHAp was thoroughly characterized using a range of instrumental techniques, including XRD for crystallinity, XPS for surface chemical analysis, FTIR and Raman spectroscopy for functional group identification, BET analysis for surface area determination, FESEM for morphological analysis, and pH_pzc_ for understanding surface charge behaviour.

The effectiveness of Ss/nHAp in adsorbing CR dye from water solutions was assessed. Batch experiments were carried out to study how different factors, such as the amount of adsorbent used, the contact time, the initial concentration of dye, and the pH level, impact the effectiveness of adsorption. The adsorption mechanism was further studied through adsorption isotherms and kinetics models to provide insights into the interaction between CR and the Ss/nHAp surface. Additionally, the regeneration potential of the synthesized Ss/nHAp was tested to assess its reusability, which plays a crucial role to determine the economic viability of the adsorbent for large-scale wastewater treatment applications.

## 2. Material and method

### 2.1 Materials

The primary material utilized in this research was seashells sourced from the species *Conus litteratus*, collected from Cox's Bazar seashore in Bangladesh. The chemicals involved included phosphoric acid (H_3_PO_4_) with 85% purity (CAS: 7664-38-2), procured from Merck KGaA, Germany, and ammonia solution (NH_4_OH; CAS: 1336-21-6), sourced Scharlau, Spain. Moreover, analytical grade CR dye was employed, acquired from Loba Chemie in India. The characteristics of CR dye are outlined in Table 1.

**Table 1:** Properties of CR dye

Property	CR dye
Chemical structure	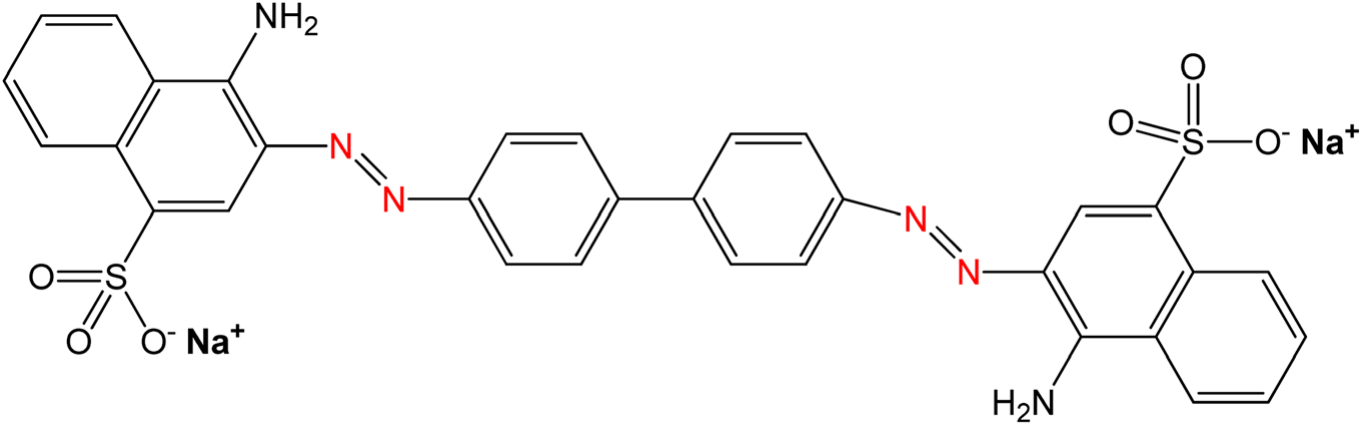
Chemical formula	C_32_H_22_N_6_Na_2_O_6_S_2_
IUPAC name	Disodium4-amino-3-[4-[4-(1-amino-4-sulfonato-naphthalen-2-yl)diazenylphenyl]phenyl]diazenyl-naphthalene-1 sulfonate
Molecular weight	696.66 g mol^−1^
CAS number	573-58-0
Appearance	Red powder
Solubility	Soluble in water
Maximum absorption wavelength (*λ*_max_)	497 nm
Dye class	Azo dye

### 2.2 Methods

#### 2.2.1 Synthesis of Ss/nHAp

**Fig. 1:**
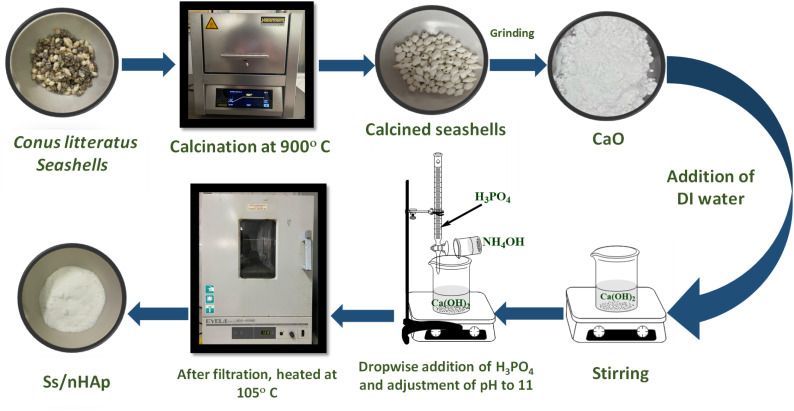
Schematic representation of Ss/nHAp synthesis by wet mixing method.

The collected *Conus litteratus* seashells were thoroughly washed with detergent to remove surface impurities followed by oven drying for several hours. Subsequently, 20 g of the cleaned seashells were calcined at a temperature of 900 °C for a period of 3 hours to transform the CaCO_3_ into CaO.^40^ After calcination, the shells were crushed using a mortar and pestle, resulting in 11.25 g of CaO. Out of this, 5.6 g of CaO was carefully added to 100 mL of deionized (DI) water to prepare a 1 M Ca(OH)_2_ solution, which was stirred constantly for 1 hour to ensure complete reaction. Following this, 100 mL of 0.6 M phosphoric acid (H_3_PO_4_), used as the phosphorus source, was gradually introduced to the mixture using a burette over a period of 30 minutes, maintaining a calcium to phosphorous ratio at 1.67. The solution was stirred continuously during this process. After the complete addition of H_3_PO_4_, the mixture was stirred for an additional 30 minutes, with a pH being adjusted to 11 using ammonia solution (NH_4_OH). The mixture was further stirred for 2 hours to facilitate the formation of HAp. Following this, it was left to settle for 18 hours. The resulting precipitate was then filtered *via* a vacuum filtration and the solid product was dried at 105 °C in an oven, producing seashell-derived nano HAp (Ss/nHAp) in powder form.^20^Fig. 1 provides a schematic illustration of the synthesis process for Ss/nHAp.

#### 2.2.2 Adsorption experiments

The elimination of CR dye from aqueous solutions was investigated by batch adsorption experiments using the synthesized Ss/nHAp as the adsorbent. In these experiments, 40 mL of a 50 ppm CR dye solution was shaken at 250 rpm using an orbital shaker at 298 K. Various parameters were tested to optimize the adsorption process, including dosage of Ss/nHAp adsorbent, contact time, pH level, and initial concentration of CR dye. Different quantities of the synthesized Ss/nHAp—specifically 0.025 g, 0.05 g, 0.1 g, 0.15 g, and 0.2 g—were tested to identify the optimal dosage for maximum CR dye removal. To examine the effect of contact time, the adsorption process was carried out for various durations: 1, 5, 10, 15, 20, 30, and 60 minutes. The influence of pH on the adsorption efficiency was assessed by adjusting the dye solution's pH to 2, 4, 6, 7, 8, 10, and 12 using 0.1 M HCl and 0.1 M NaOH. Moreover, the impact of initial dye concentration on adsorption capacity was studied by testing concentrations of 10, 20, 30, 40, 50, 60, 70, and 80 ppm. After the adsorption process, the adsorbent was separated from the solution *via* centrifugation, and the remaining dye concentration in the supernatant was determined using a Hitachi U-2910 spectrophotometer at a wavelength of 497 nm. This approach provided accurate measurement of residual dye concentration, enabling evaluation of the adsorption capacity and efficiency of the Ss/nHAp.

The quantification of CR dye adsorption onto the synthesized Ss/nHAp involved calculating the uptake of CR at various time intervals, denoted as *q*_t_ (mg g^−1^), as well as at equilibrium, represented as *q*_e_ (mg g^−1^). Additionally, the removal percentage was determined to assess the efficiency of the adsorption process, achieved through the following eqn (1)–(3).^41^1
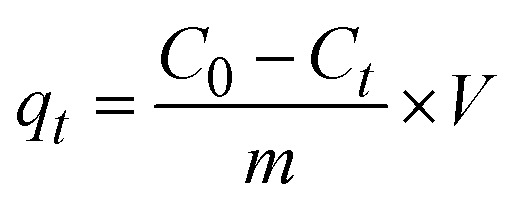
2
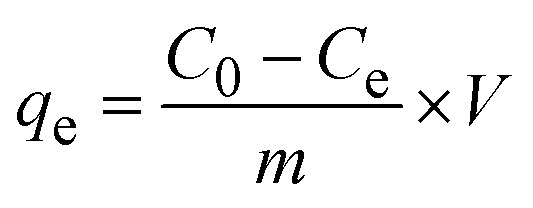
3
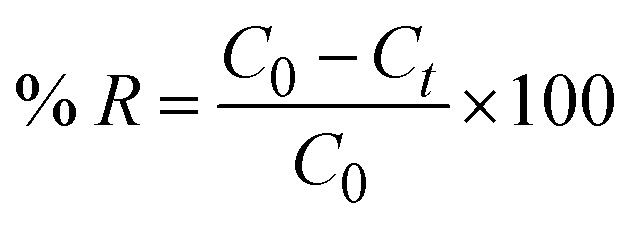
Where, *C*_0_, *C*_*t*_ and *C*_e_ represent the initial concentration, concentration at time *t* and equilibrium concentration of the CR dye in the solution (mg L^−1^), respectively. *V* denotes the volume of the solution (L) and *m* refers to the mass of the synthesized Ss/nHAp adsorbent (g).

## 3. Results and discussions

### 3.1 Characterization of Ss/nHAp adsorbent

#### 3.1.1 XRD study

**Fig. 2:**
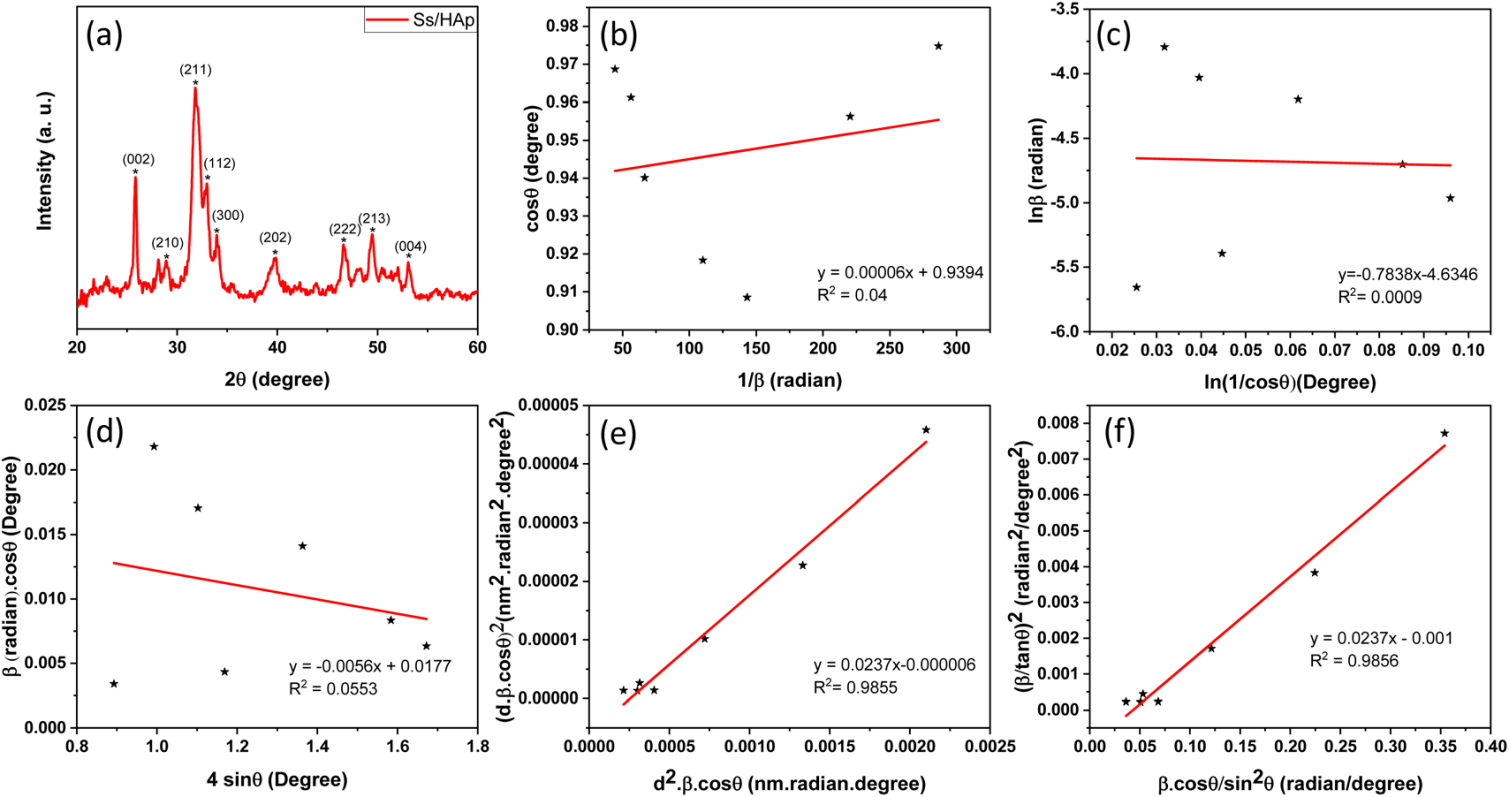
(a) XRD pattern of Ss/nHAp, linear fit plot of (a) LSLM, (b) MSM, (d) WHM, (e) SSPM, and (f) HWM for calculating crystallite size of synthesized Ss/nHAp.

The X-ray diffraction (XRD) pattern of the synthesized Ss/nHAp composite, as shown in Fig. 2(a), provides crucial insights into its crystalline structure and phase purity. The XRD analysis was carried out to determine the crystalline structure and phase purity of the synthesized hydroxyapatite. The diffraction pattern exhibited seven distinct peaks at 2*θ* values of 25.79°, 28.74°, 32.001°, 33.98°, 39.85°, 46.64°, and 49.42°, corresponding to the (002), (210), (211), (300), (202), (222), and (213) planes, respectively. The sharpest peak was observed at 32.001°, indicating a high degree of crystallinity in the (211) plane. The selected values of full width at half maximum (FWHM, *β*) for the corresponding planes and related conversions are presented in Table 2. These diffraction peaks confirm that the material possesses a hexagonal crystal structure with space group *P*6_3_/*m*, consistent with the reference JCPDS card no. 01-0721243. The analysis shows that all observed peaks are characteristic of nHAp, and no peaks corresponding to other calcium phosphate phases or impurities were found in the analysis, indicating the high purity of the synthesized material.^42^

**Table 2:** Crystallographic parameters of the synthesized Ss/nHAp based on the X-ray diffraction

2*θ* (deg)	*β* = FWHM (deg)	*θ* (deg)	cos *θ* (deg)	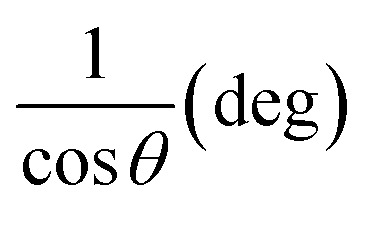	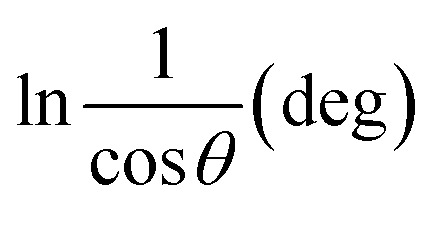	*β* = FWHM (rad)	ln *β* (rad)	4 sin *θ* (deg)	*β* (rad)·cos *θ* (deg)	*d*-Spacing (Å)
25.79	0.20	12.90	0.975	1.026	0.0255	0.0035	0.003	0.893	0.0034	3.451
28.74	1.29	14.37	0.969	1.032	0.0318	0.0225	0.023	0.993	0.0218	3.104
32.00	1.02	16.00	0.961	1.040	0.0395	0.0177	0.018	1.103	0.0171	2.795
33.99	0.26	16.99	0.956	1.046	0.0447	0.0045	0.005	1.169	0.0043	2.636
39.85	0.86	19.93	0.940	1.064	0.0618	0.0150	0.015	1.363	0.0141	2.260
46.64	0.52	23.32	0.918	1.089	0.0852	0.0091	0.009	1.584	0.0083	1.946
49.42	0.4	24.71	0.909	1.101	0.0959	0.0070	0.007	1.672	0.0063	1.843

#### 3.1.2 Crystallite size calculation using various methods

##### 3.1.2.1 Scherrer's method (SM)

The crystallite size, often referred to as the coherent domain size for a particular diffraction peak, is a key parameter in material science. In powdered samples, it generally corresponds to the grain size, whereas in polycrystalline thin films, it relates to the thickness of the film. The Scherrer equation (eqn (4)) is one of the most widely applied methods for determining this crystallite size.^43^4
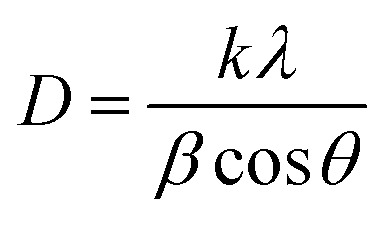
where, *D* denotes the crystallite size, *λ* represents the X-ray wavelength (0.15405 nm for Cu Kα radiation), *β* is the full width at half maximum (FWHM) of the peak in radians, *k* is the shape factor (typically set to 0.9), and *θ* is the diffraction angle. The crystallite size often calculated from the FWHM of the most intense diffraction peak.^44^ In this study, applying the Scherrer equation yielded a crystallite size of 8.13 nm for the synthesized Ss/nHAp.

##### 3.1.2.2 Scherrer equation average method (SEAM)

The Scherrer Equation Average Method (SEAM) employs all selected FWHM (*β*) values from the X-ray diffraction pattern (Fig. 2(a)) to estimate crystallite size *via* the Scherrer equation. This method calculates individual crystallite sizes for each *b* value and then averages them to obtain a representative crystallite size. The crystallite sizes calculated using SEAM, along with their average value, are shown in Table 3. By considering all or selected *β* values from prominent peaks, the estimated average crystallite size using the Scherrer equation was around 19.36 nm.

**Table 3:** Average crystallite size calculation using Scherrer equation

*β* = FWHM (rad)	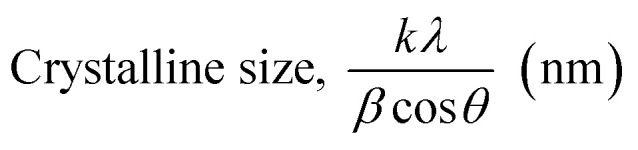	Average crystallite size (nm)
0.0035	40.75	
0.0225	6.36	
0.0177	8.13	
0.0045	31.95	19.36
0.0150	9.83	
0.0091	16.64	
0.0070	21.86	

##### 3.1.2.3 Linear straight-line method (LSLM)

To determine the crystallite size, the Linear Straight-Line Method (LSLM) is applied to the Scherrer equation. This approach involves analyzing all selected peaks and reformulating the eqn (4) into a linear form (5),^45^5
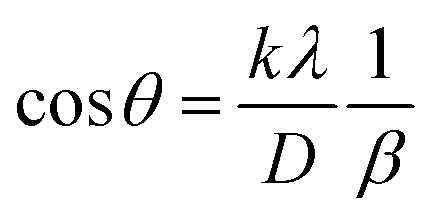


A linear relationship is observed between cos *θ* and 1/*β*, as shown in Fig. 2(b). The slope of this line, which corresponds to *kλ*/*D*, is determined to be 0.00006 (*R*^2^ = 0.04). Using this slope value, the crystallite size is calculated to be 2310.75 nm.

##### 3.1.2.4 Straight line passing the origin method (SLPOM)

The Straight Line Passing the Origin Method (SLPOM) was developed to improve the accuracy of crystallite size calculations.^45,46^ This method ensures that the linear plot intersects the origin, leading to a more reliable slope for subsequent calculations. SLPOM utilizes all data points from the linear plot (as illustrated in Fig. 2(b) and detailed in Table 4), which are introduced into the following eqn (6),6
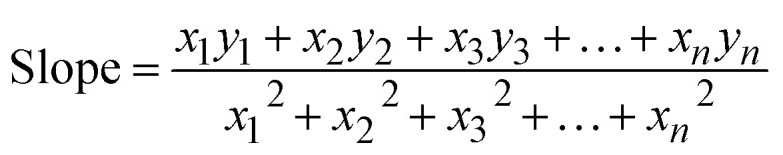


**Table 4:** The extracted values of (*x*, *y*) from Fig. 2(b)

Values of *x*	312.30	310.35	307.94	306.37	301.18	294.19	291.06
Values of *y*	0.9748	0.9687	0.9612	0.9563	0.9401	0.9183	0.9085

By applying the values of *x* and *y* to the linear regression equation, the slope was calculated to be 0.00312. This slope is equivalent to *kλ*/*D*. Consequently, the crystallite size was determined to be 44.42 nm. This method considers all selected peaks from the XRD pattern, resulting in a crystallite size that exceeds the values obtained through both the Scherrer method and the Scherrer equation average method.

##### 3.1.2.5 Monshi Scherrer method (MSM)

The Monshi Scherrer Method (MSM), also referred to as the modified Scherrer method, was developed to enhance the accuracy of crystallite size calculations.^44^ This method modifies the existing Scherrer equation. The original Scherrer equation (eqn (4)) is adapted as follows (eqn (7)),7
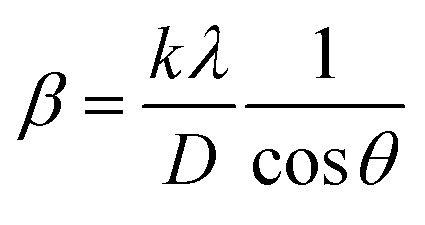
Or,8
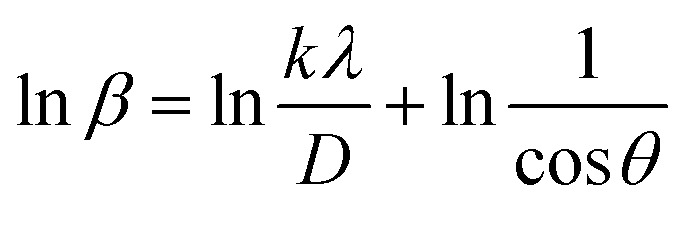


To estimate crystallite size using the MSM, a plot of ln *β vs.*
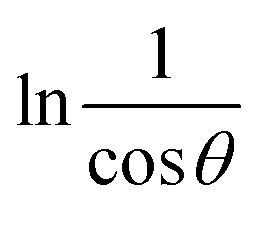
 is created. The linear fit of this plot, when compared to the straight-line equation *y* = *mx* + *c*, results in
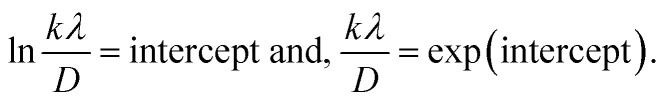


From the plot (Fig. 2(c)), the slope was found to be −0.7838 and an intercept of −4.6346 (*R*^2^ = 0.0009). Using these values, the average crystallite size was determined to be 14.28 nm, falling between the values obtained from SM and SEAM methods.

##### 3.1.2.6 Williamson–Hall method (WHM)

The Williamson–Hall Method (WHM) was employed to determine the crystallite size of the synthesized Ss/nHAp. In contrast to the Scherrer method, which only considers the impact of crystallite size on XRD peak broadening, the WHM method also accounts for strain-induced peak broadening.^47^ This method calculates both the crystallite size and the intrinsic strain, eliminating the 1/cos *θ* dependency by introducing the variation with tan *θ*.^48^ Strain arises from imperfections and distortions within the crystals.^49^ The total physical line broadening (FWHM) of an X-ray diffraction peak is expressed as a combination of size and strain effects (eqn (9))

9 FWHM_total_ = FWHM_size_ + FWHM_strain_

10
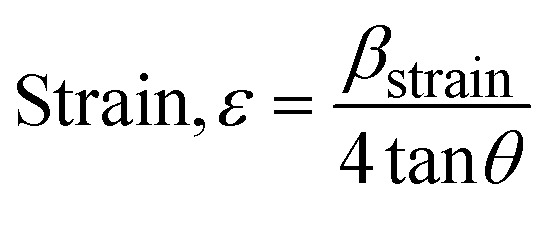


Combining eqn (7), (9) and (10) gives us eqn (11),11
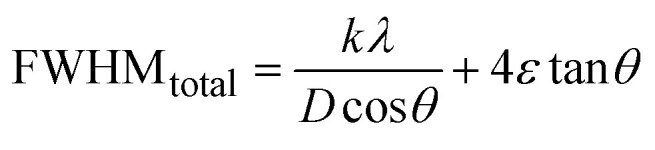


Rearranging the equation, we get eqn (12),12
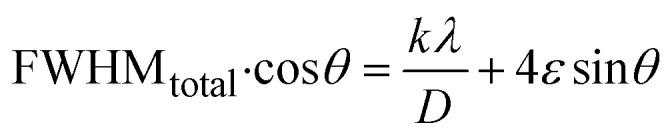


FWHM_total_·cos *θ vs.* 4 sin *θ* plot results in a linear graph (Fig. 2(d)). The slope and intercept of this line represent the strain and crystal size, respectively. From this linear fitting, the intercept was found to be 0.0177 and the slope was −0.0056 (*R*^2^ = 0.0553). Using the intercept value, the crystallite size was calculated to be 7.83 nm.

##### 3.1.2.7 Size–strain plot method (SSPM)

The SSPM separates the broadening of XRD peaks into two components: size-induced broadening, modelled as a Lorentzian function, and strain-induced broadening, modelled as a Gaussian function.^50^ This distinction allows for a more precise calculation of crystallite size and microstrain, particularly in isotropic crystal structures and low-angle reflections where precision is enhanced. The relationship is expressed by the eqn (13),

13 *β*_*hkl*_ = *β*_L_ + *β*_G_

Here, *β*_L_ denotes broadening due to finite crystallite size and *β*_G_ represents broadening due to strain.

The SSPM is represented by eqn (14) as follows,14
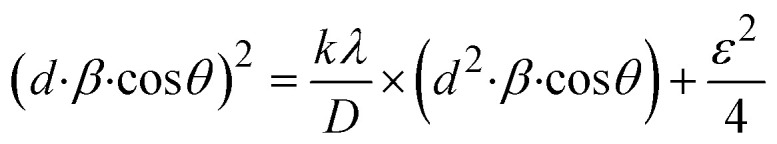


In this equation, *d* represents the lattice spacing between the (*hkl*) planes within the hexagonal structure of Ss/nHAp, calculated using eqn (15),15
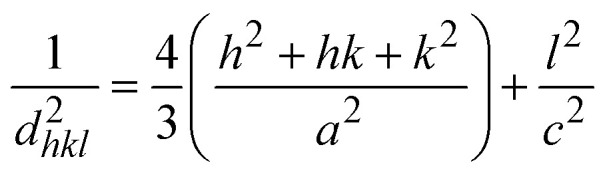


Using eqn (14), a linear plot of (*d*·*β*·cos *θ*)^2^*vs.* (*d*^2^·*β*·cos *θ*) (Fig. 2(e)) is created. From the intercept and slope of the linear fit, the intrinsic strain and crystallite size are calculated. The slope of the plot (0.0237) corresponds to 
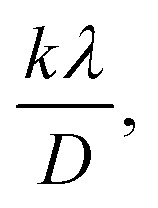
 indicating a crystallite size of 5.85 nm (*R*^2^ 0.9855).

##### 3.1.2.8 Haldar–Wagner method (HWM)

The HWM is necessary in order to determine crystallite size to the fact that the size broadening of the XRD peak profile does not follow a purely Lorentzian or Gaussian distribution. Instead, it follows a symmetric Voigt function.^51,52^ To address this complexity, HWM employs the following eqn (16),

16 *β*^2^_*hkl*_ = *β*_L_*β*_*hkl*_ + *β*^2^_G_

Here, *β*_L_ and *β*_G_ represent FWHM of the Lorentzian and Gaussian functions, respectively. HWM is more favourable compared to other methods because it specifically targets low and mid-angle peaks with minimal diffraction peak overlap. The computational equation for HWM is shown as (eqn (17)),17
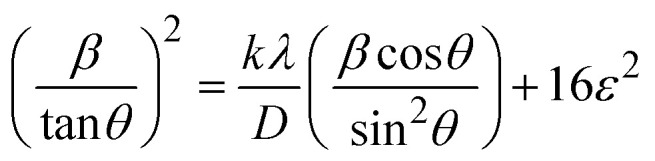

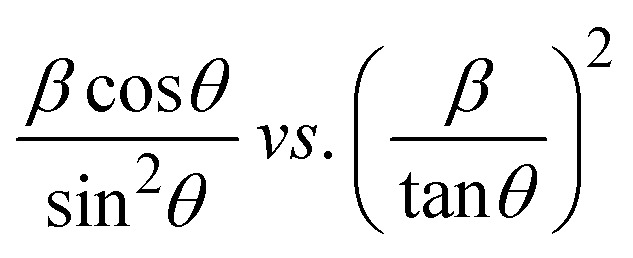
 plot results in a linear graph (Fig. 2(f)). The slope of this plot, 0.0237 with *R*^2^ value 0.9855, corresponds to 
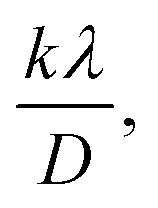
 yielding a calculated crystallite size of 5.85 nm, consistent with the SSPM results.

The crystallite size calculations for the synthesized Ss/nHAp are summarized in Table 5. Among the various methods, LSLM produced a large crystallite size of 2310.75 nm which is inconsistent with the findings of other methods and we exclude this method from our consideration.^53^ Based on the values of *R*^2^ (0.9855), SSPM and HWM are best fitted and yielded the lowest crystallite sizes of 5.85 nm, whereas the SLPOM using the sharpest peak's FWHM resulted in the highest crystallite size of 44.42 nm.

**Table 5:** The calculated crystallite size of Ss/nHAp using various methods

Sl. No.	Method	Crystallite size, *D* (nm)	*R* ^2^
01	SM	8.13	—
02	SEAM	19.36	—
03	LSLM	2310.75	0.04
04	SLPOM	44.42	—
05	MSM	14.28	0.0009
06	WHM	7.83	0.0553
07	SSPM	5.85	0.9855
08	HWM	5.85	0.9856

#### 3.1.3 Crystallographic analysis

The crystallographic parameters for synthesized Ss/nHAp were determined using XRD analysis. The material crystallizes in a hexagonal system, which is characterized by lattice values *a*, *b* and *c*. The unit cell has lattice parameters of *a* = *b* = 9.13 Å, *c* = 6.90 Å, with a volume of *V* = 498.34 Å^3^; degree of crystallinity, *X*_c_ = 0.013, micro-strain, *ε* = 0.89; and dislocation density, *δ* = 1.53 × 10^16^ lines per m^2^ were calculated using eqn (18)–(22).18
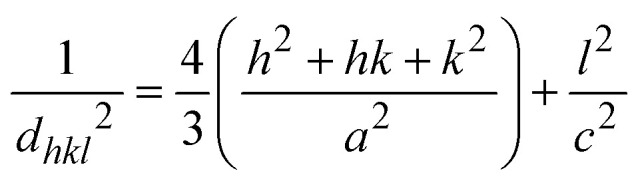
19
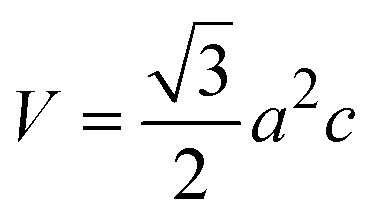
20
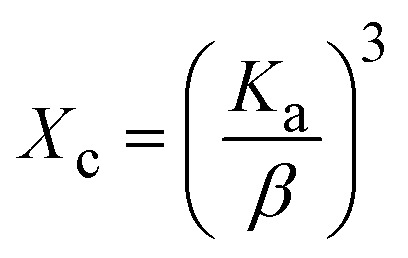
21
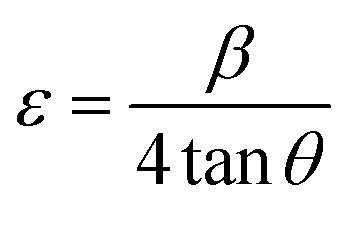
22
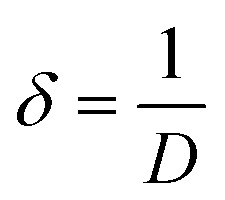
where, *d*_*hkl*_ is the interplanar spacing, *hkl* Millar indices, *K*_a_ = 0.24, *β* = FWHM, *θ* represents the diffraction angle and crystallite size is denoted by *D*.

The obtained parameters are consistent with typica values for HAp, confirming the successful synthesis of the material from seashell-derived precursors.

#### 3.1.4 XPS study

**Fig. 3:**
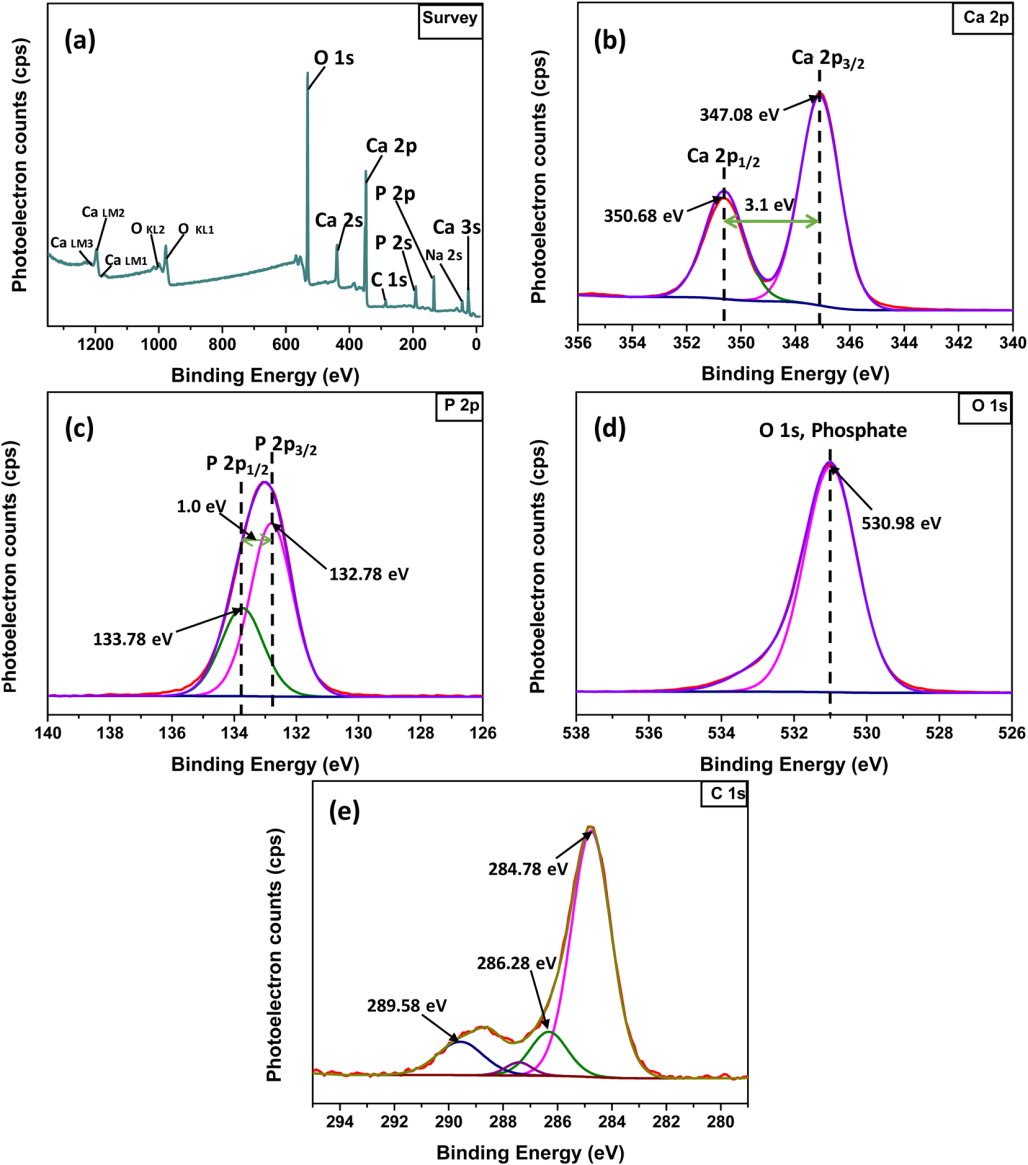
XPS analysis of Ss/nHAp: (a) survey spectra; high resolution spectra of (b) Ca 2p, (c) P 2p, (d) O 1s and (e) C 1s.

In the XPS study, the elemental composition and surface chemistry of the Ss/nHAp sample were investigated. The XPS spectrum provides detailed information on the binding energies of different elements found in the material, allowing for the identification of functional groups and confirmation of successful synthesis.^54^ The survey scan (Fig. 3(a)) reveals the existence of calcium (Ca 2p), phosphorus (P 2p), oxygen (O 1s), and carbon (C 1s). Trace amount (atomic% 0.33) of sodium was also detected as impurities which might be incorporated from the seashells. The peaks corresponding to Ca 2p, P 2p, and O 1s confirm the formation of the HAp structure.^55^ Additionally, the C 1s (atomic% 13.13) peak can be attributed to adventitious carbon present on the surface The clear peaks in the high-resolution Ca 2p spectra (Fig. 3(b)) indicate the oxidation state of Ca as Ca^2+^ in the HAp structure, with distinct signals for Ca 2p_3/2_ and Ca 2p_1/2_.^6^ The P 2p spectrum (Fig. 3(c)) exhibits a peak that corresponds to P–O bonding in the phosphate group, characteristic of HAp. In the O 1s spectrum (Fig. 3(d)), the main peak is related to the hydroxyl (OH^−^) groups and phosphate oxygen, confirming the existence of the functional groups that enhances the material's adsorption capabilities. The C 1s spectrum (Fig. 3(e)) indicates the presence of carbon-based contaminants or organic residues, often found in biogenic-derived materials, while the fitted peaks reveal different chemical environments of carbon, including C–C, C=O, and C–O, bonds.^56^

#### 3.1.5 FT-IR study

**Fig. 4:**
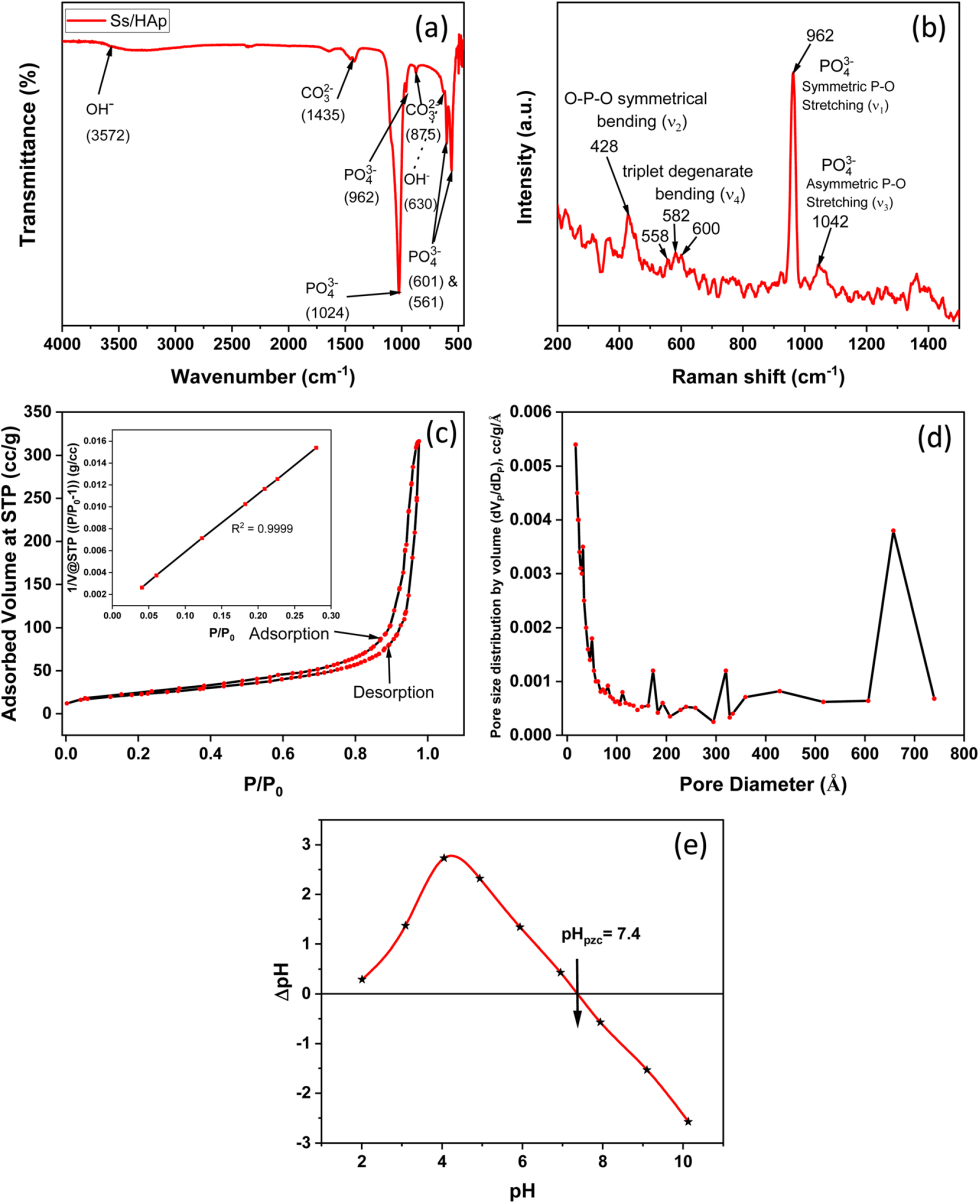
(a) FTIR spectra, (b) Raman spectra, (c) N_2_ adsorption–desorption isotherm, (d) BJH adsorption pore size distribution curve, and (e) point of zero charge (pH_pzc_) determination of Ss/nHAp.

The FTIR spectrum of the synthesized Ss/nHAp material reveals several characteristic absorption bands that represent different functional groups in the (Fig. 4(a)). The wide peak around 3572 cm^−1^ is attributed to the O–H stretching vibrations, signifying the existence of hydroxyl groups (OH^−^) in the sample.^57^ The absorption bands observed at 1435 cm^−1^ and 875 cm^−1^ are associated with the asymmetric stretching and bending vibrations of carbonate ions (CO_3_^2−^),^58^ suggesting the incorporation of these ions into the HAp structure, which is common in carbonated HAp. The significant peaks at 962 cm^−1^, 1024 cm^−1^, and 601 cm^−1^ are associated with the phosphate groups (PO_4_^3−^) which are essential components of the HAp lattice.^59^ The 630 cm^−1^ peak, also corresponds to O–H bending vibrations, further confirms the presence of hydroxyl groups in the sample.^60^ These FTIR findings show that the Ss/nHAp sample consists of a HAp phase with incorporated carbonate ions, which may affect its crystallinity and bioactivity.^10^

#### 3.1.6 Raman analysis

The Raman spectroscopy analysis of the synthesized Ss/nHAp sample, as depicted in Fig. 4(b), reveals key vibrational modes characteristic of the phosphate groups (PO_4_^3−^) within the HAp structure. The spectrum exhibits a prominent peak at 962 cm^−1^, indicating the symmetric P–O stretching mode (*ν*_1_), which is a signature of well-crystallized HAp. Additionally, the 1042 cm^−1^ peak corresponds to the asymmetric P–O stretching mode (*ν*_3_). The spectrum also shows peaks at 582 cm^−1^, 558 cm^−1^, and 600 cm^−1^, which correspond to the triplet degenerate bending modes (*ν*_4_) of the phosphate groups. Furthermore, the peak at 428 cm^−1^ corresponds to the O–P–O symmetrical bending mode (*ν*_2_). These vibrational modes confirm the presence of phosphate groups within the HAp lattice, indicating that the synthesized sample has retained its structural integrity. The sharpness and intensity of these peaks also suggest that the HAp in the Ss/nHAp sample is crystalline in nature.^55^

#### 3.1.7 Brunauer–Emmett–Teller (BET) analysis

BET analysis was employed to evaluate the specific surface area and porosity of the synthesized Ss/nHAp nanocomposite. The nitrogen adsorption–desorption isotherms, illustrated in Fig. 4(c), provide important information about the material's textural characteristics. In Fig. 4(c), the nitrogen adsorption–desorption isotherms of the HAp nanoparticles at 77 K are shown. The isotherms exhibit a Type IV classification as per IUPAC standards, with a noticeable hysteresis loop in the relative pressure (*P*/*P*_0_) range of 0.4 to 1.0. This loop is indicative of mesoporous structures, which are pores with diameters between 2 and 50 nm.^61^

The initial steep rise in nitrogen adsorption at low relative pressures (*P*/*P*_0_ < 0.1) can be attributed to monolayer-multilayer adsorption on the surface of the HAp nanoparticles. As the relative pressure increases, the adsorption curve transitions to a nearly linear region, reflecting multilayer adsorption. The presence of the hysteresis loop suggests capillary condensation within the mesopores.^62^ The surface area determined by the BET method, utilizing the linear section of the adsorption isotherm (usually at *P*/*P*_0_ = 0.05–0.3), was measured to be 81.05 m^2^ g^−1^.

The pore size distribution was analyzed using the Barrett–Joyner–Halenda (BJH) method, as depicted in Fig. 4(d). The analysis indicates a predominant pore size range of 2 to 50 nm, confirming the mesoporous nature of the HAp nanoparticles. The average pore diameter is calculated to be 24.05 nm, with a total pore volume of 0.49 cm^3^ g^−1^. These comprehensive analyses illustrate the significant mesoporosity and high surface area of the synthesized HAp nanoparticles, highlighting their suitability for advanced applications in biomedical and industrial fields.

#### 3.1.8 Point of zero charge analysis

Fig. 4(e) represents the determination of the point of zero charge (pH_pzc_) of the material, a crucial parameter in surface chemistry. The pH_pzc_ is the pH at which the material's surface has no net electrical charge, indicating an equal number of positive and negative charges on the surface.^63^ The point where the difference in pH (ΔpH) before and after equilibration is zero corresponds to the pH_pzc_. For this material, the pH_pzc_ is observed at pH 7.4. At this pH, the surface has a neutral charge. Below pH 7.4, the material surface becomes positively charged due to protonation of surface groups, making it more likely to attract negatively charged species (anions). Conversely, above pH 7.4, the surface becomes negatively charged due to deprotonation of surface groups, leading it to attract positively charged species (cations).^63^ Understanding the pH_pzc_ is important in predicting the material's behaviour in different pH environments, especially for applications involving adsorption.

#### 3.1.9 FESEM and EDX study

**Fig. 5:**
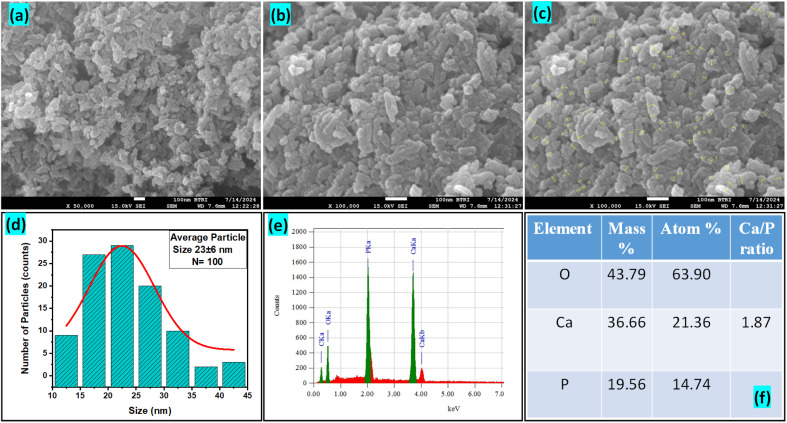
(a)–(c) FESEM images, (d) particle size distribution histogram, (e) EDX spectra, and (f) elemental composition of Ss/nHAp.

The FESEM images presented in Fig. 5(a–c) reveal the morphology of the synthesized Ss/nHAp nanoparticles. The particles appear to have a predominantly agglomerated, irregular spindle like structure, rather than a spherical shape. The surface morphology suggests good homogeneity in particle distribution, as indicated by the high-magnification images. The particle size distribution (Fig. 5(d)) indicates an average size of particle, 23 ± 6 nm. The EDX analysis (Fig. 5(e)) confirms the elemental composition of the sample, with significant peaks attributed to O, Ca, and P, further validating the formation of Ss/nHAp. The mass percentages of O, Ca, and P are 43.79%, 36.66%, and 19.56%, respectively (Fig. 5(f)). The calculated Ca/P ratio of 1.87 is close to the stoichiometric ratio of 1.67 for hydroxyapatite, indicating a slight calcium excess that may contribute to variations in crystallinity or defect structures within the material.^64^

### 3.2 Adsorption studies

#### 3.2.1 Effect of adsorbent dose

**Fig. 6:**
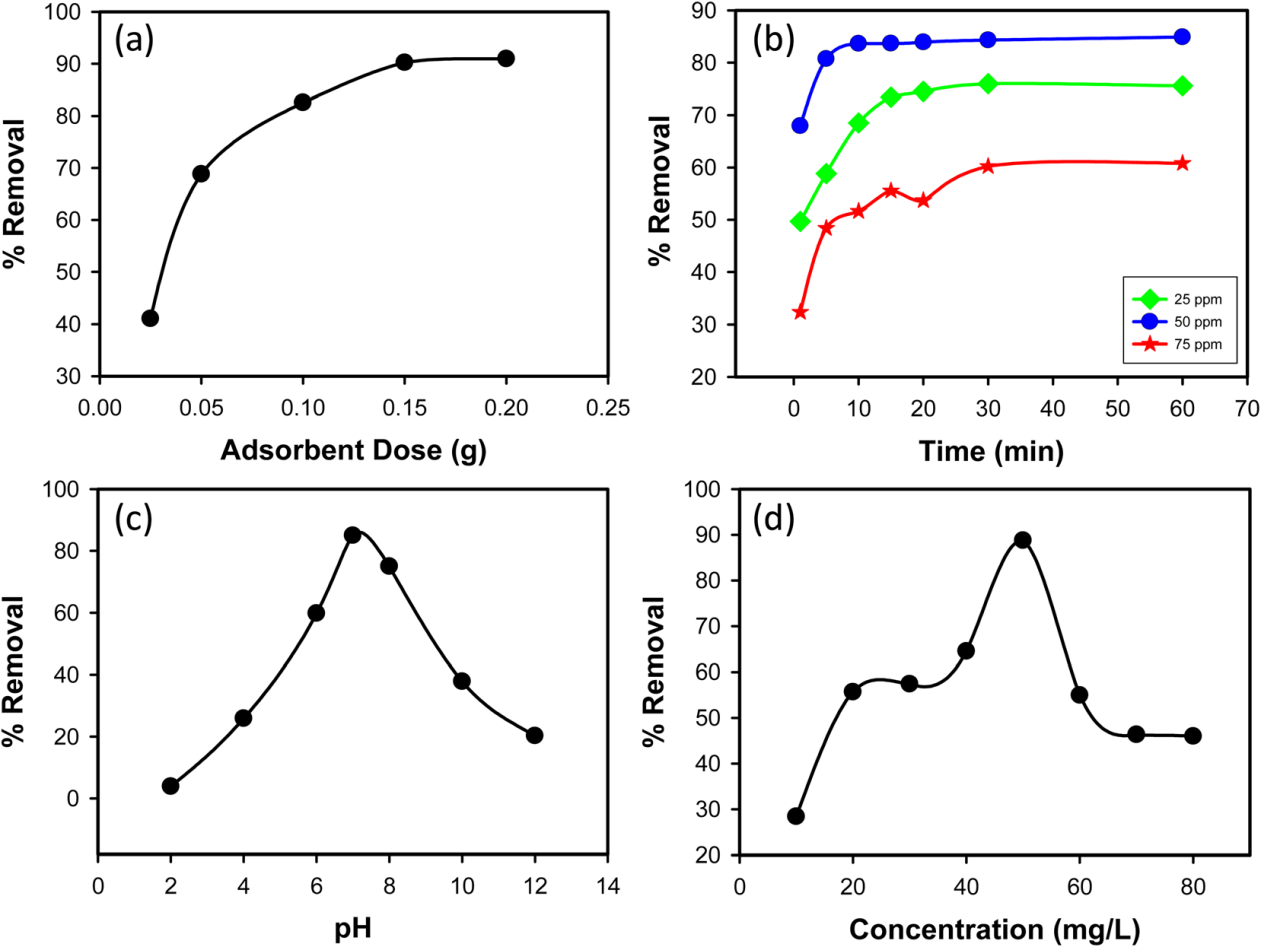
(a) Effect of adsorbent dose (*C*_0_ = 50 mg L^−1^, time = 30 min, pH = 7, shaking speed = 200 rpm); (b) effect of contact time (*C*_0_ = 25, 50 & 75 mg L^−1^, Ss/nHAp dose = 0.1 g, pH = 7, shaking speed = 200 rpm); (c) effect of pH (*C*_0_ = 50 mg L^−1^, Ss/nHAp dose = 0.1 g, time = 10 min, shaking speed = 200 rpm); and (d) effect of initial CR concentration (Ss/nHAp dose = 0.1 g, time = 10 min, pH = 7, shaking speed = 200 rpm).

The impact of adsorbent dosage on CR dye removal efficiency was examined by varying the amount of synthesized Ss/nHAp composite used in the adsorption process. Doses of 0.025 g, 0.05 g, 0.1 g, 0.15 g, and 0.2 g were tested with an initial CR dye concentration of 50 mg L^−1^. As shown in Fig. 6(a), the percentage removal of CR dye increased with increasing adsorbent dosage. At a dose of 0.025 g, the removal efficiency was 41.07%, significantly increasing to 68.83% at a dose of 0.05 g. Further increases in dosage to 0.1 g, 0.15 g, and 0.2 g led to removal efficiencies of 82.57%, 90.24%, and 90.97%, respectively. This trend indicates that higher amounts of Ss/nHAp composite provide more adsorption sites, enhancing CR dye removal. Based on these results, an optimal adsorbent dose of 0.1 g was determined, providing a high removal efficiency of 82.57% while balancing adsorbent usage.

#### 3.2.2 Effect of contact time

The impact of contact time on CR dye removal efficiency was investigated over a range of 1 to 60 minutes for three initial dye concentrations: 25, 50, and 75 mg L^−1^. As shown in Fig. 6(b), the highest removal efficiency was observed at an initial concentration of 50 mg L^−1^. The removal efficiency of CR dye increased with contact time for all initial concentrations. For the 25 mg L^−1^ concentration, the removal efficiency increased from 49.68% at 1 minute to 75.56% at 60 minutes. For the 50 mg L^−1^ concentration, the removal efficiency increased from 67.95% at 1 minute to 84.89% at 60 minutes. For the 75 mg L^−1^ concentration, the removal efficiency increased from 32.40% at 1 minute to 60.80% at 60 minutes. Among the three initial concentrations, the 50 mg L^−1^ solution exhibited the highest removal efficiency across all contact times. Based on these results, an optimal contact time of 10 minutes was determined. At this time, the removal efficiencies for 25, 50, and 75 mg L^−1^ were 68.54%, 83.64%, and 51.65%, respectively. This optimal time balances removal efficiency and operational convenience, making it a practical choice for the adsorption process using the synthesized Ss/nHAp composite.

#### 3.2.3 Effect of pH

The removal efficiency of CR dye, an anionic dye, was tested across a pH range of 2 to 12 (Fig. 6(c)). At pH 2.0, the removal efficiency was low at 3.91%, due to high hydrogen ion concentration competing with dye molecules for adsorption sites and protonation of both the adsorbent surface and the –NH_2_ groups on the dye, reducing interaction. As the pH increased, the removal efficiency improved, peaking at 85% at pH 7.0, where deprotonation of the adsorbent surface enhanced adsorption. Beyond pH 7.0, the efficiency decreased, with 75% removal at pH 8.0 and dropping further to 37.86% at pH 10.0 and 20.27% at pH 12.0. This decline is due to increased hydroxide ions competing with dye molecules for adsorption sites and reduced dye solubility. The optimal pH for adsorption was determined to be 7.0, providing the highest removal efficiency. This pH level optimizes the surface charge conditions of the Ss/nHAp composite for effective dye removal.

#### 3.2.4 Effect of initial concentration of dye

The impact of initial CR dye concentration on removal efficiency was investigated using concentrations ranging from 10 mg L^−1^ to 80 mg L^−1^. As shown in Fig. 6(d), removal efficiency initially increased with dye concentration. At a low concentration of 10 mg L^−1^, removal efficiency was 28.39%. As the concentration increased to 20 mg L^−1^ and 30 mg L^−1^, removal efficiency significantly improved to 55.68% and 57.43%, respectively. The highest removal efficiency of 88.78% was observed at a concentration of 50 mg L^−1^. This increase in removal efficiency with higher concentrations can be attributed to a greater driving force for mass transfer due to the higher concentration gradient. However, beyond the 50 mg L^−1^ concentration, removal efficiency began to decline. At 60 mg L^−1^, efficiency dropped to 54.97%, and further decreased to 46.33% at 70 mg L^−1^ and 45.98% at 80 mg L^−1^. This decrease at higher concentrations can be attributed to saturation of active sites on the adsorbent, where available adsorption sites become insufficient to accommodate excess dye molecules. Based on these observations, the optimal initial concentration for the adsorption process was determined to be 50 mg L^−1^, providing the highest removal efficiency. This concentration ensures effective adsorbent utilization while maintaining a high removal rate of CR dye from the aqueous solution.

### 3.3 Kinetics study

**Fig. 7:**
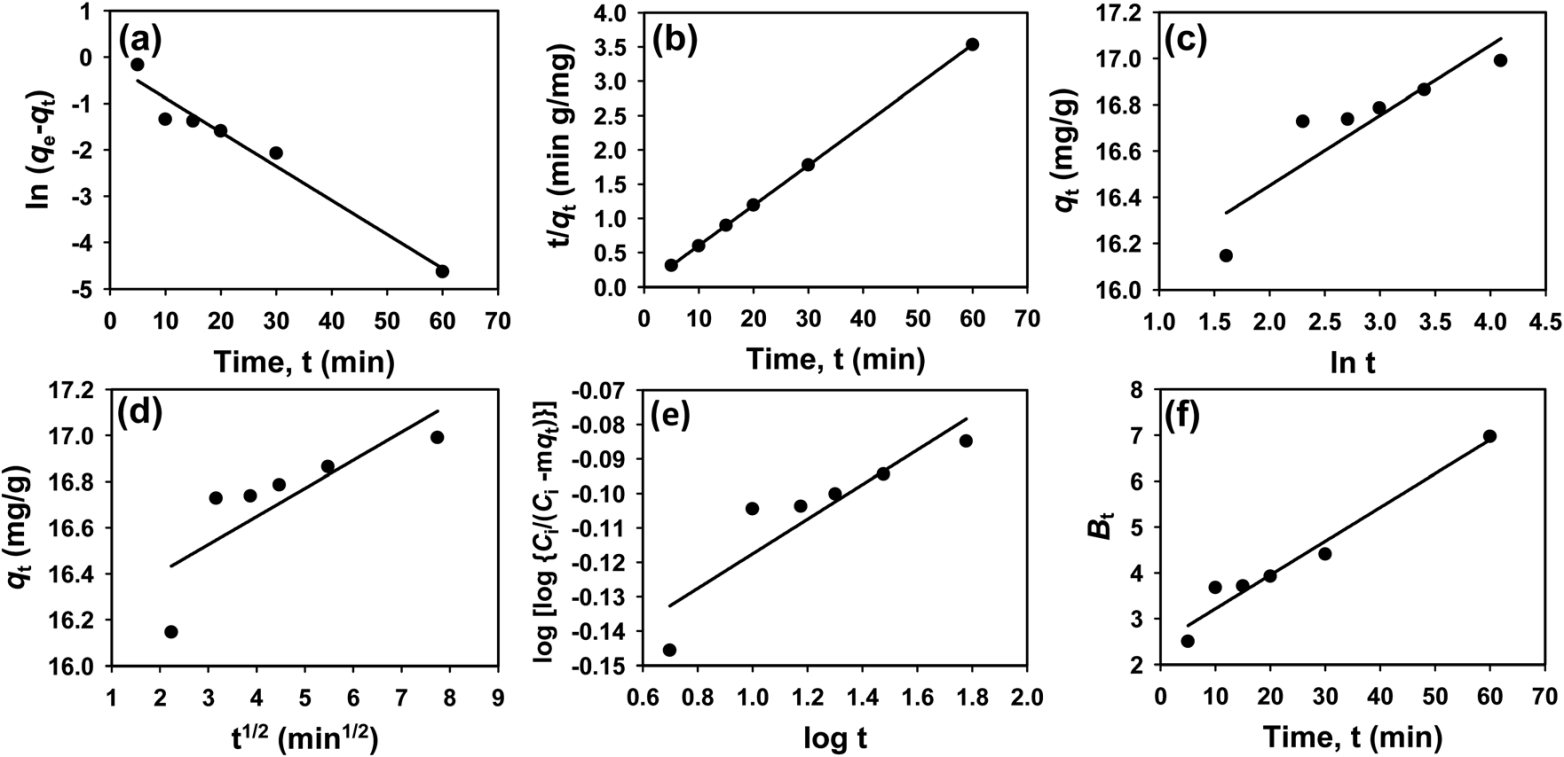
Adsorption kinetics for CR removal by Ss/nHAp: (a) pseudo first order; (b) pseudo second order; (c) Elovich; (d) intra-particle diffusion; (e) Bangham and (f) Boyd kinetic model.

The kinetics of CR dye adsorption onto the synthesized Ss/nHAp composite were studied to understand the rate and mechanism of the adsorption process. Batch adsorption experiments were conducted at an initial dye concentration of 50 mg L^−1^, using an adsorbent dose of 0.1 g, and varying the contact time from 1 to 60 minutes. The adsorption kinetics were evaluated using several models, each offering insights into different aspects of the process. The pseudo-first-order (Fig. 7(a)) and pseudo-second-order (Fig. 7(b)) models examine the rate of adsorption based on the availability of active sites, with the latter providing evidence for chemisorption as the dominant mechanism through its best fit to the data. The Elovich model (Fig. 7(c)) highlights surface heterogeneity and describes the declining adsorption rate over time due to the saturation of active sites. The intraparticle diffusion model (Fig. 7(d)) helps identify whether diffusion within pores limits the rate of adsorption and provides insights into boundary layer effects through the intercept of its linear plot. The Bangham model (Fig. 7(e)) focuses on pore diffusion and multi-stage adsorption, offering a deeper understanding of transport mechanisms within the adsorbent. Lastly, the Boyd model (Fig. 7(f)) determines whether the rate-controlling step involves external film diffusion or intraparticle diffusion. Eqn (23)–(28) show the linearized equations for these models,^65^

23 ln(*q*_e_ − *q*_*t*_) = ln *q*_e_ − *k*_1_*t*

24
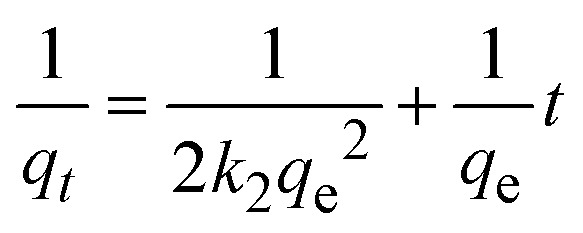
25
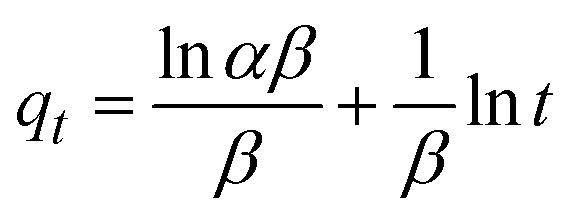

26 *q*_*t*_ = *k*_i_*t*^0.5^ + *C*

27
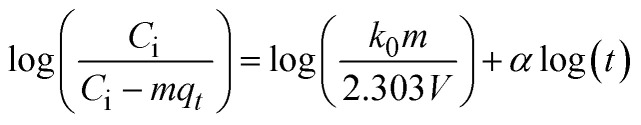
28
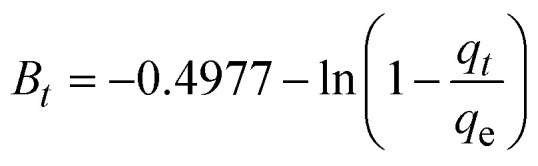
where, *q*_e_ (mg g^−1^) and *q*_*t*_ (mg g^−1^) represent the quantity of dye adsorbed at equilibrium and at time *t*, respectively. *k*_1_ (min^−1^) denotes the rate constant of the pseudo-first-order adsorption; *k*_2_ (g mg^−1^ min^−1^) is the rate constant of the pseudo-second-order adsorption; *α* (mg g^−1^ min^−1^) and *β* (g mg^−1^) are constants in the Elovich equation; *k*_i_ (mg g^−1^ min^−0.5^) is the intraparticle diffusion rate constant; *C* (mg g^−1^) is the intercept in the Weber–Morris model indicating the boundary layer effect; *C*_0_ (mg L^−1^) is the initial concentration of the dye solution, *m* (g L^−1^) is the mass to volume ratio of adsorbent; *k*_0_ (mg g^−1^) and *α* are constants in the Bangham equation; and *B*_*t*_ is the Boyd kinetic constant at time *t*.

Table 6 presents the parameters derived from the kinetic models, along with their respective *R*^2^ values: 0.9615, 1.0000, 0.8014, 0.6545, 0.8124, and 0.9615. Among these models, the Ho's pseudo-second-order kinetic model exhibited the best fit, as indicated by the highest *R*^2^ value of 1.0000 (Fig. 7(b)). This suggests that the adsorption process is primarily chemisorption, involving valence forces through the sharing or exchange of electrons between the adsorbent and dye molecules.^66^ The pseudo-second-order model assumes that the rate-limiting step is chemical adsorption, similar to covalent bonding. The high *R*^2^ value demonstrates that this model accurately predicts the adsorption capacity and effectively represents the overall behaviour of the adsorption process compared to the other models.

**Table 6:** Parameters derived from kinetic models

Kinetic model	Curve fitting	Parameters	Values
Pseudo-first order	Linear	*q*_e_ (mg g^−1^)	0.8636
		*k*_1_ (min^−1^)	0.0736
		*R* ^2^	0.9615
**Pseudo-second order**	**Linear**	***q***_**e**_ **(mg g^−^** ^**1**^ **)**	**17.065**
		***k***_**2**_ **(g mg^−^** ^**1**^ **min^−^** ^**1**^ **)**	**9.6099**
		***R*** ^**2**^	**1.0000**
Elovich	Linear	*α* (mg g^−1^.min)	3.0360
		*β* (g mg^−1^)	3.2992
		*R* ^2^	0.8014
Intra-particle diffusion	Linear	*C* (mg g^−1^)	16.160
		*k*_i_ (mg g^−1^ min^−0.5^)	0.1220
		*R* ^2^	0.6545
Bangham		*R* ^2^	0.8124
Boyd	Linear	*R* ^2^	0.9615

### 3.4 Isotherm study

**Fig. 8:**
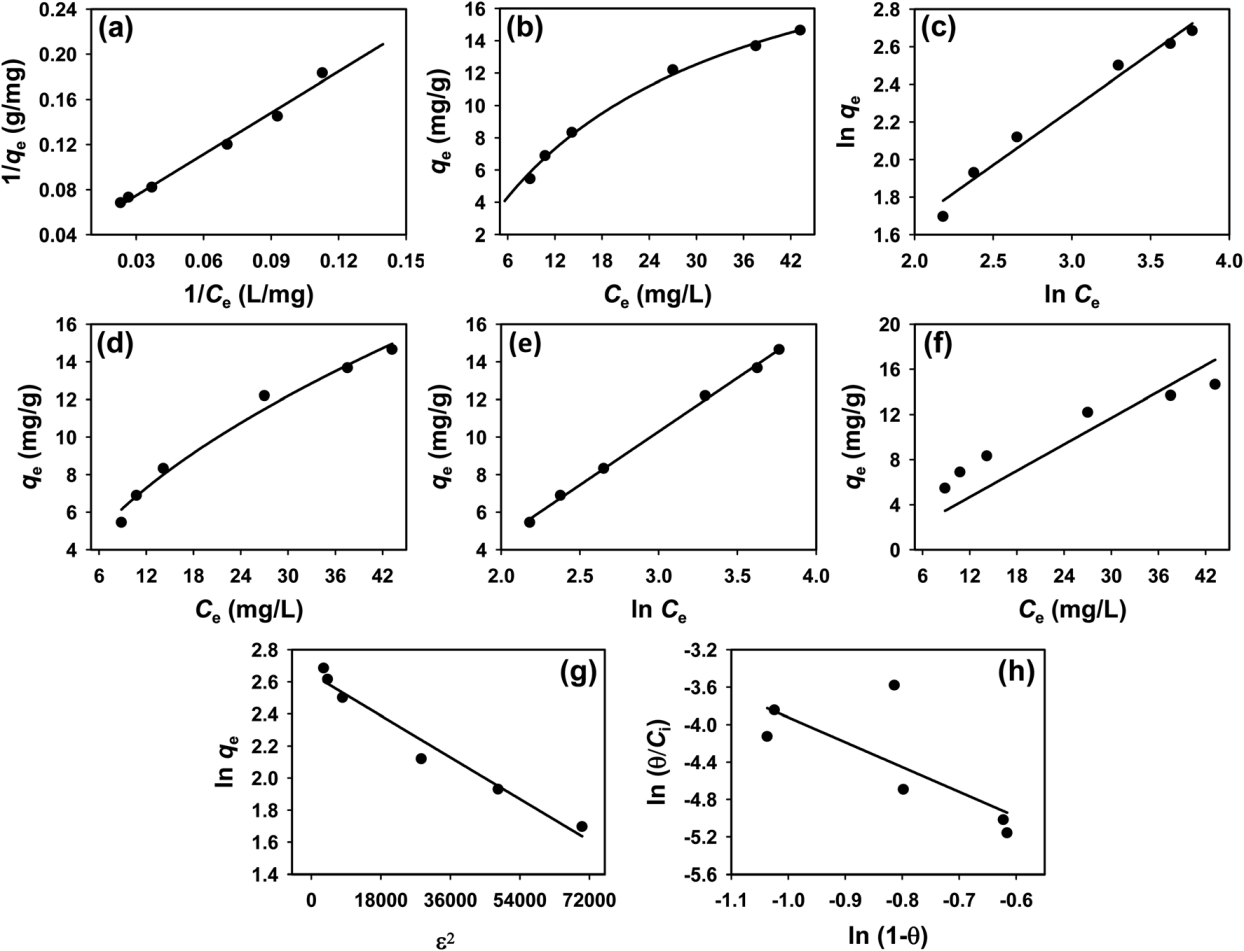
Adsorption isotherms for CR removal by Ss/nHAp: (a) linear, (b) non-linear Langmuir isotherm model; (c) linear, (d) non-linear Freundlich isotherm model; (e) Temkin; (f) Henry; (g) Dubinin–Radushkevich and (h) Flory–Huggins isotherm model.

To understand the interaction between the CR dye and the synthesized Ss/nHAp composite, various isotherm models were applied: Langmuir linear (Fig. 8(a)) and non-linear (Fig. 8(b)), Freundlich linear (Fig. 8(c)) and non-linear (Fig. 8(d)), Temkin (Fig. 8(e)), Henry (Fig. 8(f)), Dubinin–Radushkevich (D–R) (Fig. 8(g)), and Flory–Huggins (Fig. 8(h)). These models are represented by eqn (29)–(36).^67,68^29
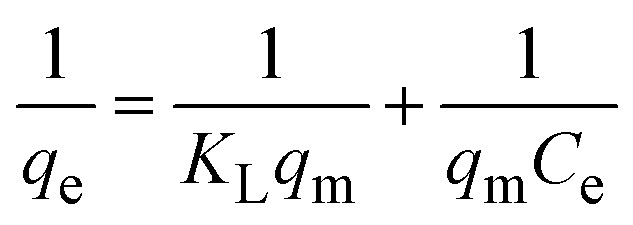
30
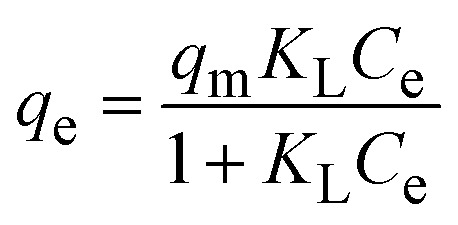
31
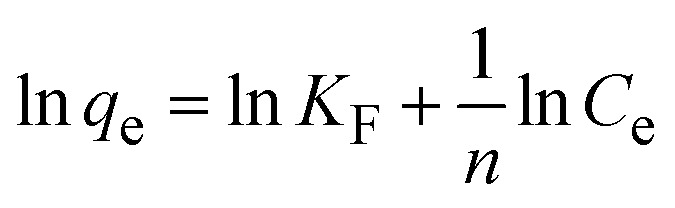

32 *q*_e_ = *K*_F_*C*^1/*n*^_e_

33
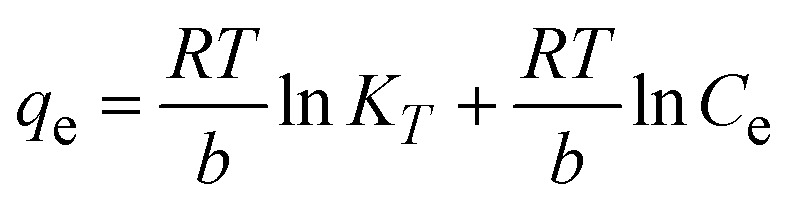

34 *q*_e_ = *K*_H_*C*_e_

35 ln *q*_e_ = ln *q*_m_ − *βε*^2^

36
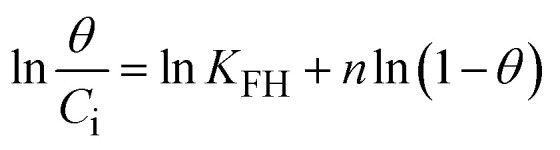


The data obtained from these isotherm models are detailed in Table 7, with *R*^2^ values of 0.8807, 0.9955, 0.9789, 0.9835, 0.9982, 0.6205, 0.9676, and 0.5704, respectively. Isotherm studies reveal that the adsorption of CR dye onto the Ss/nHAp composite is best described by the non-linear Langmuir, Freundlich, and Temkin isotherm models, as evidenced by their *R*^2^ values (0.9955, 0.9835, and 0.9982, respectively). The Langmuir model suggests monolayer adsorption on a homogenous surface,^69^ while the Freundlich model indicates a heterogeneous surface with varied energy sites.^70^ The Temkin model highlights the importance of adsorbate/adsorbate interactions in the adsorption process. Conversely, the Henry and Flory–Huggins models showed poor fits, indicating they do not adequately describe the adsorption behaviour for this system. The D–R model's good fit suggests that pore-filling mechanisms^71^ are also significant in the adsorption process. Based on the isotherm analysis, the adsorption process predominantly follows the Langmuir model, indicating monolayer coverage on a uniform surface (*q*_max_ 23.94 mg g^−1^). However, the contribution of Freundlich and Temkin models suggests some heterogeneity and molecular interactions.

**Table 7:** Parameters derived from isotherm models

Isotherm model	Curve fitting	Parameters	Values
Langmuir	Linear	*q*_m_ (mg g^−1^)	26.316
		*K*_L_ (L mg^−1^)	0.0311
		*R* ^2^	0.8807
	Non-linear	***q***_**m**_ **(mg g^−^** ^**1**^ **)**	**23.939**
		***K***_**L**_ **(L mg^−^** ^**1**^ **)**	**0.0366**
		***R*** ^**2**^	**0.9955**
Freundlich	Linear	*K*_F_ (mg g^−1^) (L mg^−1^)^1/*n*^	1.6225
		*N*	1.68
		***R*** ^**2**^	0.9789
	Non-linear	*K*_F_ (mg g^−1^) (L mg^−1^)^1/*n*^	1.8063
		*n*	1.78
		***R*** ^**2**^	**0.9835**
Temkin	Linear	*K*_T_ (L mg^−1^)	0.3039
		*b* (J mol^−1^)	435.39
		***R*** ^**2**^	**0.9982**
Henry	Linear	*K*_H_ (L g^−1^)	0.3898
		*R* ^2^	0.6205
Dubinin–Radushkevich	Linear	*q*_m_ (mg g^−1^)	14.141
		*β* (mol^2^ kJ^−2^)	1.44 × 10^−8^
		*E* (kJ mol^−1^)	5884.59
		*R* ^2^	0.9676
Flory–Huggins	Linear	*K*_FH_ (L g^−1^)	0.0014
		Δ*G*° (kJ mol^−1^)	−16.29
		*R* ^2^	0.5704

### 3.5 Thermodynamics study

To determine the thermodynamic feasibility of the adsorption process, the Gibbs free energy change (Δ*G*°) was calculated using equilibrium constants from various isotherm models (Table 8). The eqn (37) was employed for this calculation,

37 Δ*G*° = −*RT* ln *K*

where *R* is the universal gas constant (8.314 J mol^−1^ K^−1^) and *T* is the temperature in Kelvin.

**Table 8:** Gibbs free energy derived from various isotherm models

Isotherm	Fitting	Constant, *K* (L mg^−1^)	Constant, *K* (L mol^−1^)	Gibbs free energy, Δ*G*° (J mol^−1^)	Gibbs free energy, Δ*G*° (kJ mol^−1^)
Langmuir	Linear	0.0311	21635.4	−24731	−24.73
	Non-linear	0.0366	25497.8	−25138	−25.14
Freundlich	Linear	1.6225	1130366.8	−34532	−34.53
	Non-linear	1.8063	1258376.9	−34798	−34.80
Temkin	Linear	0.3039	211699.7	−30382	−30.38

The negative values of Δ*G*° for all the isotherm models represents that the adsorption process is spontaneous at 298 K temperature. The more negative the Δ*G*° value, the more thermodynamically favourable the adsorption process.^72^ Among the models, the Freundlich non-linear model showed the most negative Δ*G°* value of −34.80 kJ mol^−1^, followed closely by the Freundlich linear model with −34.53 kJ mol^−1^. These values suggest a highly favourable and efficient adsorption process according to the Freundlich isotherm, which indicates a heterogeneous adsorption surface with varying adsorption sites. The Temkin model, with a Δ*G*° value of −30.38 kJ mol^−1^, also demonstrate a significant degree of spontaneity, emphasizing the role of adsorbate–adsorbent interactions during the adsorption process. The Langmuir models, both linear and non-linear, had Δ*G*° values of −24.73 kJ mol^−1^ and −25.14 kJ mol^−1^ respectively. While these values still indicate spontaneity, they are less negative compared to the Freundlich and Temkin models, suggesting that the adsorption process according to the Langmuir model, which assumes monolayer adsorption on a homogeneous surface, is also favourable but less so compared to a heterogeneous surface scenario as described by the Freundlich model. Therefore, the thermodynamic analysis confirms that the adsorption of CR dye onto Ss/nHAp composite is a spontaneous and thermodynamically favourable process across all isotherm models tested, with the Freundlich model providing the most negative Δ*G*° values, indicating the highest degree of spontaneity.

### 3.6 XPS and FTIR analyses for CR loading mechanism

**Fig. 9:**
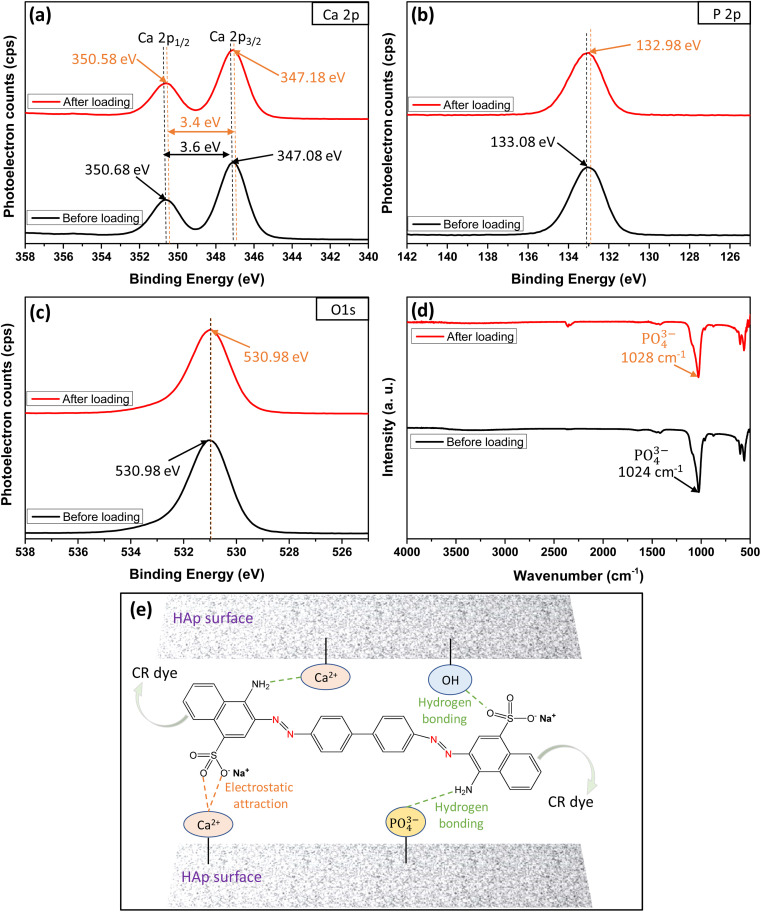
XPS core level spectra of (a) Ca 2p, (b) P 2p, (c) O 1s; (d) FTIR spectra before and after loading of CR dye on Ss/nHAp; and (e) mechanism of CR dye adsorption on Ss/nHAp.

The XPS and FTIR analyses provide valuable insights into the mechanism of CR dye adsorption onto Ss/nHAp. In the XPS spectra, there are notable shifts in the binding energies of Ca 2p (Fig. 9(a)) and P 2p (Fig. 9(b)), indicating interactions between CR and the Ss/nHAp surface. Specifically, the Ca 2p binding energy gap decreases from 3.6 eV to 3.4 eV after CR loading, which suggests an electrostatic attraction between Ca^2+^ ions attached to Ss/nHAp surface and the negatively charged sulfonate groups (–SO^−^_3_) of CR (Fig. 9(e)). The P 2p binding energy shift from 133.08 eV to 132.98 eV further points to the involvement of phosphate (PO^3−^_4_) groups in hydrogen bonding with the amine groups (–NH_2_) of CR (Fig. 9(e)). However, the O 1s (Fig. 9(c)) peak does not show significant changes, indicating that the oxygen-related species in Ss/nHAp remain unaffected during the adsorption process, and no direct interaction with CR occurs at the oxygen sites.

FTIR analysis (Fig. 9(d)) supports these observations, particularly in the shift of the characteristic PO^3−^_4_ band. The PO^3−^_4_stretching band around 1024 cm^−1^ exhibits a shift to 1028 cm^−1^ after CR adsorption, suggesting hydrogen bonding between PO^3−^_4_ and CR's –NH_2_. The absence of significant shifts in the OH-related peaks in the FTIR spectrum, consistent with the O 1s XPS data, confirms that hydroxyl groups do not play a crucial role in CR adsorption. These results demonstrate the effective removal of CR *via* surface interactions between the CR dye and Ss/nHAp, with no direct involvement of oxygen-related species on the Ss/nHAp surface.

### 3.7 Regeneration studies

**Fig. 10:**
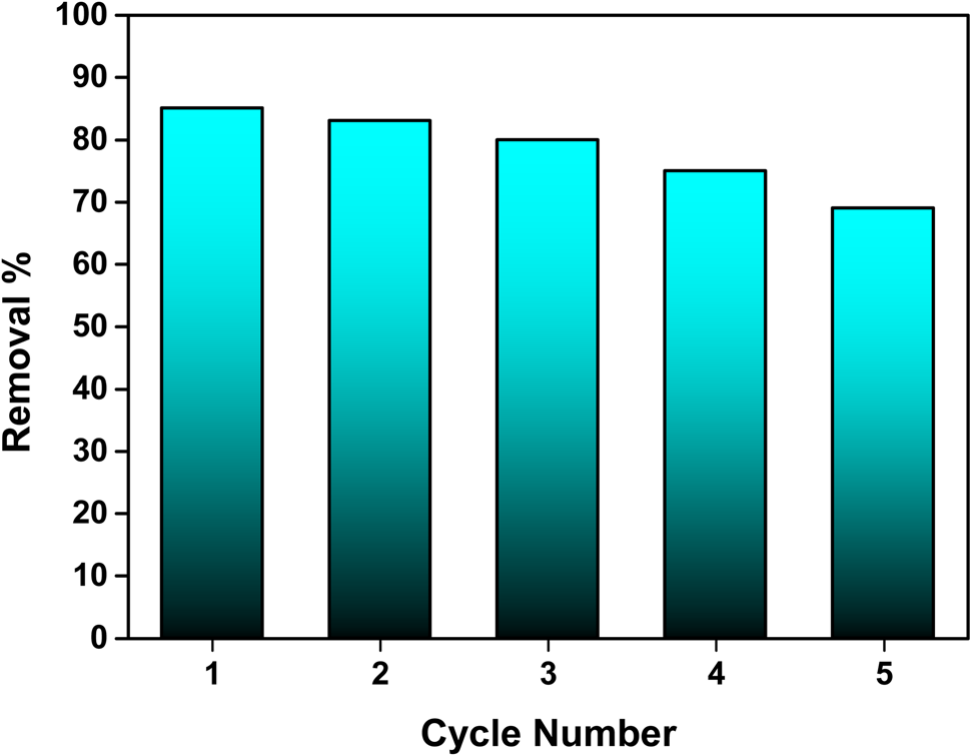
Stability and reusability assessment of the Ss/nHAp adsorbent.

The regeneration potential of the synthesized hydroxyapatite (Ss/nHAp) was evaluated through multiple adsorption–desorption cycles to assess its reusability and cost-effectiveness in the removal of CR dye (Fig. 10). In the first adsorption cycle, Ss/nHAp achieved 85% removal efficiency of the CR dye, indicating its strong affinity and high capacity for dye adsorption. After the initial cycle of CR dye adsorption, the adsorbent was regenerated using a systematic process. Initially, the used adsorbent was rinsed with DI water to eliminate physically adsorbed dye molecules from the surface. It was then treated with 0.5 M NaOH, which acted as a desorption agent to break the chemical interactions between the CR molecules and the active sites on the Ss/nHAp.^6^ Following this, the adsorbent was washed again with DI water to eliminate any NaOH residue, ensuring no chemical contamination for the next adsorption cycle. Finally, the adsorbent was oven-dried at 105 °C to restore its dry state, which is optimal for further adsorption experiments. In subsequent cycles, the elimination efficacy of CR dye was 83% in the second cycle, followed by 80%, 75%, and 69% in the third, fourth, and fifth cycles, respectively. This gradual decline in adsorption efficacy might be attributed to several factors, primarily the potential loss of adsorbent during the washing and regeneration steps. Each cycle involves handling the material extensively, and minor losses of adsorbent during washing could result in fewer available active sites in the subsequent cycles, reducing the overall adsorption capacity.

### 3.8 Comparison of Ss/nHAp with other adsorbents

A comparison of Ss/nHAp with other adsorbents to eliminate CR dye is shown in Table 9. Despite its lower adsorption capacity of 24 mg g^−1^, Ss/nHAp demonstrates a significant advantage in achieving equilibrium in just 10 minutes with a small dose of Ss/nHAp, 2.5 g L^−1^ at neutral pH. The adsorption process adheres to pseudo-second-order kinetics and aligns with several isotherm models, such as Temkin, Langmuir, Freundlich, and Dubinin–Radushkevich (D–R). In contrast, other adsorbents like HAp/chitosan and zein/HAp exhibit higher adsorption capacities (769 mg g^−1^ and 416.7 mg g^−1^, respectively), but require considerably longer equilibrium times and larger doses. This highlights Ss/nHAp as a practical and efficient adsorbent for rapid CR dye removal, particularly in scenarios where short processing times and lower doses are desired.

**Table 9:** Adsorption capacities and adsorption parameters used for removal of CR

Adsorbent	Adsorption capacity (mg g^−1^)	Equilibrium time (min)	Dose (g L^−1^)	pH	Kinetics	Isotherm	Reference
Calcium-rich fly ash	9.41	50	10	5	Pseudo-second order	Freundlich & D–R	73
HAp/chitosan	769	480	50	5	Pseudo second order	Langmuir	74 and 75
Zein/HAp	416.7	20	5.5	5.8	Pseudo second order	Langmuir	75 and 76
Bael shell carbon	98.03	100	1	3	Pseudo second order	Freundlich	77
Egg shell-derived HAp	9.64	20	5	7.5	Pseudo second order	Langmuir	6
Ss/nHAp	24	10	2.5	7	Pseudo second order	Langmuir	This study
						Temkin	
						Freundlich and D–R	

## 4. Conclusion

Hydroxyapatite (Ss/nHAp) derived from seashells (*Conus litteratus*) was successfully synthesized and utilized as an adsorbent to remove CR dye from aqueous solutions. The material was characterized using various instrumental techniques, including XRD, XPS, FTIR, Raman, BET, FESEM, and pH_pzc_. These analyses confirmed its structural integrity and suitability for adsorption purposes. Batch adsorption experiments optimized the conditions for CR dye removal, establishing that the best parameters included 0.1 g of adsorbent, a contact time of 10 minutes, a temperature of 25 °C, and an initial dye concentration of 50 ppm at neutral pH. Under these optimized conditions, Ss/nHAp exhibited a notable adsorption efficiency, with a maximum capacity of 24 mg g^−1^, demonstrating a strong affinity for CR dye. Adsorption behaviour followed both Langmuir and Freundlich isotherms, indicating a combination of monolayer and multilayer adsorption on varied surface sites. Kinetic analysis indicated a pseudo-second-order model, pointing to chemisorption as the primary mechanism. Regeneration studies confirmed the adsorbent's reusability over several cycles, with a minor decline in efficiency due to material loss during washing. The findings emphasize the potential of Ss/nHAp as a cost-effective, sustainable, and eco-friendly adsorbent for the removal of anionic dyes from industrial wastewater. Future studies should focus on scaling up the production process, investigating its efficacy for removing a broader range of pollutants, and improving the material's durability for long-term applications. This work contributes to advancing sustainable wastewater treatment technologies by utilizing renewable waste resources, offering an environmentally responsible solution for pollution remediation.

## Data availability

All the data used in this article will be available in the repository of BCSIR (Bangladesh Council of Scientific and Industrial Research), Bangladesh.

## Author contributions

Md Sohag Hossain – methodology, data collection, data analysis, writing – original draft; Md Sahadat Hossain – data collection; Samina Ahmed – supervision, funding acquisition; Mashrafi Bin Mobarak – conceptualization, methodology, data analysis, writing – review & editing, project administration, supervision.

## Conflicts of interest

There are no conflicts to declare.
